# Protocol for enhanced proliferation of human pluripotent stem cells in tryptophan-fortified media

**DOI:** 10.1016/j.xpro.2022.101341

**Published:** 2022-04-22

**Authors:** Kotaro Kameda, Shota Someya, Jun Fujita, Keiichi Fukuda, Shugo Tohyama

**Affiliations:** 1 Department of Cardiology, Keio University School of Medicine, Shinjuku, Tokyo 160-8582, Japan

**Keywords:** Cell culture, Metabolism, Stem Cells

## Abstract

We describe a protocol for the efficient culture of human pluripotent stem cells (hPSCs) by supplementing conventional culture medium with L-tryptophan (TRP). TRP is an essential amino acid that is widely available at an affordable cost, thereby allowing cost-effective proliferation of hPSCs compared to using a conventional medium alone. Here, we describe the steps for enhanced proliferation of hPSCs from dermal fibroblasts or peripheral blood cells, but the protocol can be applied to any hPSCs.

For complete details on the use and execution of this protocol, please refer to Someya et al. (2021).

## Highlights

• Preparation for TRP-fortified medium from conventional hPSC maintenance medium
• Replacement of conventional hPSC maintenance medium by TRP-fortified medium
• Evaluation for proliferation and pluripotency of hPSCs cultured in TRP-fortified medium

## Graphical abstract

**Figure:**
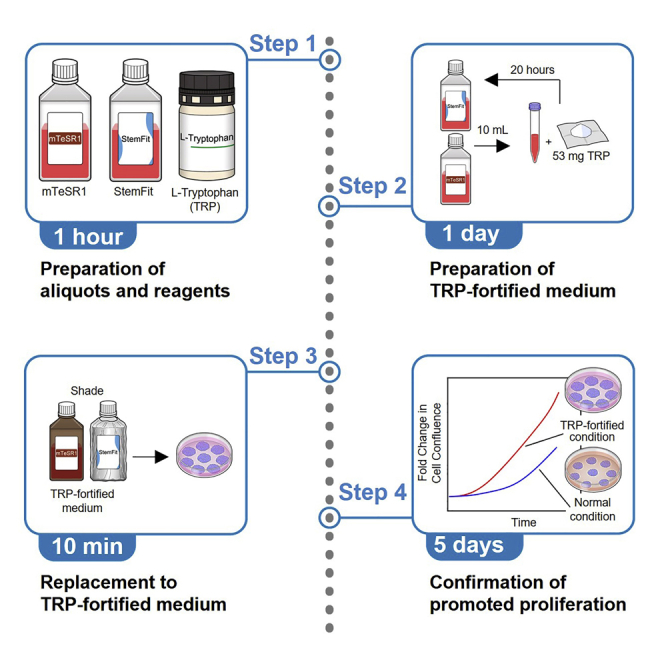

## Before you begin

This protocol describes the specific steps for hPSC culture, which have been validated in several cell lines. We applied this protocol to the human induced pluripotent stem cell (hiPSC) lines (253G4 and FfI14s04).

hPSCs were obtained from the Center for iPS Cell Research and Application, Kyoto University (Kyoto, Japan) and the WiCell Research Institute (Wisconsin, USA). Their utilization and distribution met the relevant institutional and government guidelines and regulations. We used humidified 5% CO_2_ incubators at 37°C for cell culture. All cell lines were regularly tested for contamination, including mycoplasma, and all culture procedures were performed using sterile techniques to prevent contamination. All processes within the protocol were performed in a biosafety cabinet (Class II, Type A1), according to the relevant guidelines and regulations. Regular karyotyping is highly recommended for ruling out gross chromosomal instability. We typically passaged hPSCs weekly, with the medium changed every 48 h, unless specified.

### Preparation of culture medium with TRP supplementation

**Timing: 30 min**

1. TRP (53 mg) should be directly added to an aliquot of approximately 50 mL drawn from an original 500 mL mTeSR1 bottle containing 400 mL of mTeSR1 basal medium called Solution A without the 5× Supplement.
  a. Measure TRP carefully using a clean laboratory spatula and a calibrated analytical measuring scale.
  b. Fill the tube or container thoroughly with an aliquot of mTeSR1 while keeping the original bottle covered with a shading sheet (aluminum foil can be used for practical purposes) to avoid light exposure.

***Note:*** The amount of mTeSR1 used as a solvent can be increased to assist in dissolving TRP.

***Alternatives:*** Solution A from StemFit AK02N medium can be used as a substitute for mTeSR1 basal medium.

2. Place the aliquot and the original bottle in a refrigerator at 4°C, allowing the dissolution of TRP in the dark.
3. Thaw mTeSR1 5× Supplement at 4°C.

**Pause point:** TRP and mTeSR1 5× supplement, typically, dissolve within 24 h at 4°C.

4. Carefully remove the shading sheet to observe the solution for any solute residues present. If the residues are observed, store the aliquot in a refrigerator for a longer period until dissolution. Troubleshooting 1.
5. Once a TRP is in solution, the mixture should be added back to the original mTeSR1 bottle using a 0.22-μm sterile filter.
6. Add thawed mTeSR1 5× supplement to the solution to complete the preparation of TRP-fortified mTeSR1 solution.

***Alternatives:*** For fortifying StemFit AK02N medium, thawed solution B should be added to the StemFit AK02N medium.

7. Store the bottle at 4°C until use.

***Note:*** Ensure that all resources used in this protocol are within their expiry dates. Once opened, StemFit AK02N or mTeSR1 medium should be used within two weeks.

**CRITICAL:** We strongly suggest avoiding UV light and heat exposure to prevent TRP degradation, as well as FGF2 destabilization, in StemFit AK02N or mTeSR1 medium (Bellmaine et al., 2020; Igarashi et al., 2007).

### Preparation of Matrigel-coated dishes or plates

**Timing: 30 min**

8. Dispense a mixture of thawed Matrigel and DMEM/F-12 (1:60 dilution) into dishes or plates. The detailed method follows the manufacturer’s instructions. Troubleshooting 2.
  a. Thaw an aliquot containing Matrigel on ice.
  b. Add the required amount of Matrigel from an aliquot to cold DMEM/F-12.
  c. Dispense 10 mL of the solution into a 10 cm dish, or 2 mL per well for a 6-well plate, and gently swirl to cover the entire surface with Matrigel before incubation.
9. Incubate dishes or plates in a sterile hood or refrigerator.

**Pause point:** Matrigel-coated dishes or plates are ready to be used after incubation at room temperature (approximately 21°C) for 1 h, or overnight incubation in a refrigerator at 4°C. They can be stored at 4°C for up to one week. For incubation or storage in a refrigerator, they should be covered with clean plastic wrap (or equivalent) to prevent spillage. Avoid tilting to prevent uneven surface distribution of Matrigel.

**Figure 1:**
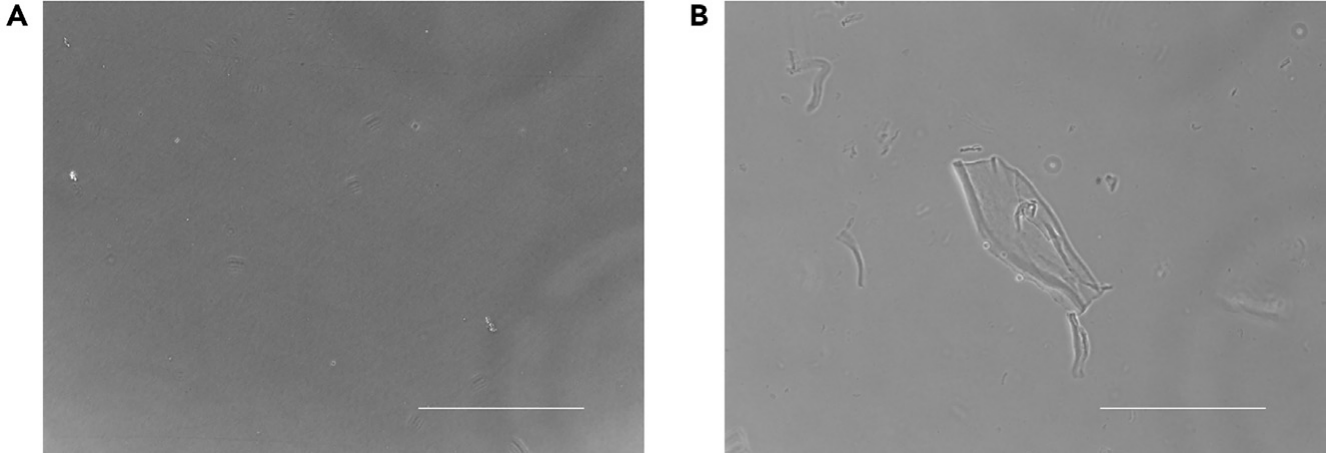
Accidental aggregation of Matrigel (A and B) If Matrigel coating was successful, (A) only small aggregates were observed. However, if the Matrigel coating failed, (B) large aggregates were frequently observed (B). Scale bar, 1 mm.

***Note:*** Prior to aspiration, Matrigel-coated dishes should be observed for aggregates (Figure 1), which may indicate that the storage, thawing, or dispensing process was inappropriately performed or at a temperature above an acceptable range.

***Alternatives:*** iMatrix-511 is a potential alternative to Matrigel, and, if used, should be diluted in D-PBS(-) at a mixing ratio of 50 μL:10 mL before performing step 8c.

## Key resources table

**Table:** 

REAGENT or RESOURCE	SOURCE	IDENTIFIER
**Chemicals, peptides, and recombinant proteins**		

mTeSR1, cGMP	STEMCELL Technologies	Cat#85850
StemFit AK02N	AJINOMOTO	Cat#RCAK02N
L-Tryptophan	Sigma-Aldrich	Cat#T8941
Matrigel Growth Factor Reduced Basement Membrane Matrix	Corning	Cat#354230
iMatrix 511	Nippi	Cat#892012
DMEM/F-12	Thermo Fisher Scientific	Cat#11320
CultureSure Y-27632	FUJIFILM Wako Pure Chemical	Cat#034-24024
DMSO	Sigma-Aldrich	Cat#D2650
D-PBS(-)	FUJIFILM Wako Pure Chemical	Cat#045-29795
TrypLE Select	Thermo Fisher Scientific	Cat#12563011
STEM-CELLBANKER GMP-grade	Zenogen Pharma	Cat#CB045
FBS	BioWest	Cat#S1560
Anti-REA Control (S)-PE, human (used at 1:10)	Miltenyi Biotec	Cat#130-104-612
Anti-SSEA-4-PE, human (used at 1:10)	Miltenyi Biotec	Cat#130-100-635
Anti-TRA-1-60-PE, human (used at 1:10)	Miltenyi Biotec	Cat#130-100-350
4% Paraformaldehyde	Muto Pure Chemicals	Cat#33111
Triton X-100	Sigma-Aldrich	Cat#9036-19-5
ImmunoBlock	KAC	Cat#CTKN001
Anti Nanog, host: rabbit, polyclonal IgG (used at 1:100 dilution)	Abcam	Cat#ab21624
Oct-3/4 Antibody (C-10), anti-human, host: mouse, monoclonal IgG2b (used at 1:200 dilution)	Santa Cruz Biotechnology	Cat#sc-5279
Anti-Stage-Specific Embryonic Antigen-4 Antibody, clone MC-813-70, anti-human/mouse, host: mouse, monoclonal IgG3 (used at 1:200 dilution)	Merck	Cat#MAB4304
Goat anti-Mouse IgG (H+L) Cross-Adsorbed Secondary Antibody, Alexa Fluor 594 (used at 1:1,000 dilution)	Thermo Fisher Scientific	Cat#A-11005
Donkey anti-Rabbit IgG (H+L) Highly Cross-Adsorbed Secondary Antibody, Alexa Fluor 594 (used at 1:1,000 dilution)	Thermo Fisher Scientific	Cat#A-21207
Hoechst 33342, Trihydrochloride, Trihydrate - 10 mg/mL Solution in Water (used at 1:2,000 dilution)	Thermo Fisher Scientific	Cat#H3570

**Experimental models: Cell lines**		

hiPSC line	Kyoto University	253G4
hiPSC line	Kyoto University	FfI14s04

**Software and algorithms**		

Kaluza Analysis Software	Beckman Coulter	n/a
FlowJo	BD Biosciences	n/a
BZ-X Analyzer	KEYENCE	n/a
BZ-X Viewer	KEYENCE	n/a
Excel for Microsoft 365	Microsoft	n/a

**Other**		

2 mL Cryogenic Vial	Sumitomo Bakelite	Cat#MS-4603
Pipet-Aid Xpress	Drummond Scientific	Cat#4-000-135
Millex-GV Syringe Filter Unit	Merck Millipore	Cat#SLGVR33RS
Vi-CELL XR Cell Viability Analyzer System	Beckman Coulter	Cat#383721
BICELL Bio Freezing Vessel	Nihon Freezer	Cat#BICELL
IncuCyte ZOOM	Essen Bioscience	N/A
Gallios Flow Cytometer	Beckman Coulter	Cat#773231AD
Falcon 5 mL Round Bottom Polystyrene Test Tube, with Cell Strainer Snap Cap	Corning	Cat#352235
Fluorescence microscope	KEYENCE	Cat#BZ-X710
Laboratory refrigerator (4°C)	N/A	N/A
Laboratory freezer (−30°C)	N/A	N/A
Laboratory freezer (−80°C)	N/A	N/A
Ultra-low temperature freezer (−150°C)	N/A	N/A
37°C water bath	N/A	N/A
Biosafety cabinet (Class II, Type A1)	N/A	N/A
Centrifuge	N/A	N/A
CO_2_ incubator (5% CO_2_, 37°C)	N/A	N/A
Inverted microscope	N/A	N/A
Cryotube freezing box	N/A	N/A
Laboratory spoon	N/A	N/A
Analytical balance	N/A	N/A
Polypropylene microtube	N/A	N/A
Aluminum foil	N/A	N/A
Plastic wrap	N/A	N/A

## Materials and equipment

**Table:** Cell culture

Reagent	Final concentration	Amount
mTeSR1 Basal Medium	4.325 × 10^-2^ mM (TRP)	400 mL
mTeSR1 5× Supplement	n/a	100 mL
L-Tryptophan	5.19 × 10^-1^ mM (TRP)	53 mg
**Total**	**5.536 × 10**^**-1**^**mM (TRP)**	**500 mL**

***Note:*** TRP-fortified mTeSR1 medium should be thoroughly covered with a material that has a UV light-shielding properties and can be stored at 4°C for up to 2 weeks. TRP can be stored at room temperature and used before its expiry date.

***Alternatives:*** StemFit AK02N medium can be used as an alternative to mTeSR1.

**Table:** 

Reagent	Final concentration	Amount
Matrigel Growth Factor Reduced Basement Membrane Matrix	7–10 mg/mL (Matrigel)	1 mL
DMEM/F-12	n/a	59 mL
**Total**	**116.7–166.7 μg/mL (Matrigel)**	**60 mL**

***Note:*** A Matrigel bottle should be thawed on ice, divided into aliquots (e.g., 1.5 mL) using microtubes, and stored at −30°C for up to 2 years to prevent thaw-freeze cycles.

***Note:*** Aliquots should be thawed on ice. Dilution with cold DMEM/F-12 should be performed promptly to avoid gel aggregation.

**Table:** 

Reagent	Final concentration	Amount
CultureSure Y-27632	n/a	1 mg
DMSO	n/a	295.6 μL
**Total**	**10 mM (Y-27632)**	**295.6 μL**

***Note:*** Y-27632 stock solution should be divided into small aliquots using microtubes and stored at −30°C for up to 1 year. Avoid multiple freeze–thaw cycles. DMSO can be stored at room temperature and used before expiry date.

**CRITICAL:** DMSO readily penetrates the skin and may cause irritation to the eyes, skin, and respiratory tract. Appropriate protection must be provided during handling.

**Table:** Flow cytometry

Reagent	Final concentration	Amount
FBS	100%	0.3 mL
D-PBS	n/a	14.7 mL
**Total**	**2% (FBS)**	**15 mL**

***Note:***Key resources table for phycoerythrin (PE)-conjugated antibodies used for flow cytometry. Prepare 2% FBS solution in each experiment.

### Immunocytochemistry

***Note:*** Prepare each solution in each experiment.

**Table:** 

Reagent	Final concentration	Amount
Triton X-100	100%	0.01 mL
D-PBS	n/a	10 mL
**Total**	**0.1% (Triton X-100)**	**10.01 mL**

**Table:** 

Reagent	Final Concentration	Amount
Anti Nanog Host: rabbit, Isotype: IgG	n/a	0.02 mL
ImmunoBlock	n/a	1.98 mL
**Total**	**1:100 dilution (NANOG antibody)**	**2 mL**

**Table:** 

Reagent	Final concentration	Amount
Donkey anti-Rabbit IgG (H+L) Secondary Antibody	n/a	0.002 mL
ImmunoBlock	n/a	1.998 mL
**Total**	**1:1,000 dilution (donkey anti-rabbit IgG antibody)**	**2 mL**

***Note:*** The donkey anti-rabbit IgG secondary antibody corresponds to the NANOG primary antibody.

**Table:** 

Reagent	Final concentration	Amount
Oct-3/4 Antibody (C-10) Host: mouse, Isotype: IgG2b	n/a	0.01 mL
ImmunoBlock	n/a	1.990 mL
**Total**	**1:200 dilution (Oct-3/4 antibody)**	**2 mL**

**Table:** 

Reagent	Final concentration	Amount
Anti-Stage-Specific Embryonic Antigen-4 Host: mouse, isotype: IgG3	n/a	0.01 mL
ImmunoBlock	n/a	1.99 mL
**Total**	**1:200 dilution (SSEA4 antibody)**	**2 mL**

**Table:** 

Reagent	Final concentration	Amount
Goat anti-Mouse IgG (H+L) Cross-Adsorbed Secondary Antibody	n/a	0.001 mL
ImmunoBlock	n/a	0.999 mL
**Total**	**1:1,000 dilution (goat anti-mouse IgG antibody)**	**1 mL**

***Note:*** The goat anti-mouse IgG secondary antibody was paired with the OCT-4 and SSEA4 primary antibodies listed above.

**Table:** 

Reagent	Final concentration	Amount
Hoechst 33342	10 mg/mL	0.001 mL
D-PBS	n/a	1.999 mL
**Total**	**0.02 mg/mL**	**2.0 mL**

## Step-by-step method details

### Initial culture of hPSCs

**Timing: 45–60 min**

In this step, hPSCs are thawed from cryotube containing frozen hPSCs to commence culture of hPSCs.

1. Place the required amount of refrigerated mTeSR1 solution in a 50 mL conical tube covered by a shading sheet at room temperature (approximately 21°C) for approximately 30–60 min to warm.

***Note:*** 10 mL of medium is required for a 10 cm dish, or 2 mL of medium per well in a 6-well plate. Moreover, 10 mL of medium is required for pelleting thawed cells and 10 mL is required for the initial culture of iPSCs.

2. Thaw an aliquot of 10 mM of Y-27632 diluted with DMSO.
3. Add 10 mM Y-27632 to the required amount of normal mTeSR1 solution (1:1,000 dilution) to prepare an mTeSR1 solution containing 10 μM Y-27632.
4. When the incubation and warming steps are completed, the dish or plate is tilted to aspirate and remove the excess Matrigel solution in the bottom corner.
5. Once the Matrigel solution was removed, mTeSR1 solution with Y-27632 should be dispensed to prevent desiccation.
6. Thaw hPSCs were stored in cryotubes at −150°C in a 37°C water bath.
7. After thawing, move the cryotube to a hood, and transfer the solution into a conical tube containing 10 mL of normal mTeSR1 with Y-27632, using a sterile 1,000-μL tip attached to a P1000 pipette.
8. Centrifuge the tube for 3 min at 300 × *g.*
9. The supernatant should be aspirated carefully to prevent suction of the cell pellet, 10 mL of normal mTeSR1 with 10 μM Y-27632 was added, and the cells were gently stirred, preferably by multiple bouts of suction and discharge of contents using an electronic pipette controller with a sterile serological pipette.

**CRITICAL:** Do not repeat pipetting more than ten times, as this may cause cell death.

10. Transfer an aliquot of the solution onto dishes or plates with the planned cell density. In this experiment, cells were seeded at approximately 3.4 × 10^3^ cells per cm^2^.

***Note:*** The volume to be transferred is in accordance with the specified split ratio of the cells, which should be selected based on the desired passaging day. If you wish to passage cells sooner, then the split ratio should be higher to allow the cells to reach sufficient confluence on the planned day of passage. In our suggestion, if you passage in 2 or 4 days, seed cells with a density of 1.7 × 10^4^ or 3.4 × 10^3^ per cm^2^, respectively. To avoid direct seeding from a high-density suspension, which may cause more variation in the initial distribution, with 6-well plates, 500 μL of medium per well was added before dispensing the diluted solution with 1.5 mL of medium containing a designated number of cells per well.

11. Place the dishes or plates in an incubator at 37°C and gently move them side-to-side to evenly spread the cells without contamination.

***Note:*** We recommend initial seeding of hPSCs in a conventional setting without TRP, fortifying with TRP, to allow cells to grow in an accustomed medium environment.

**Figure 2:**
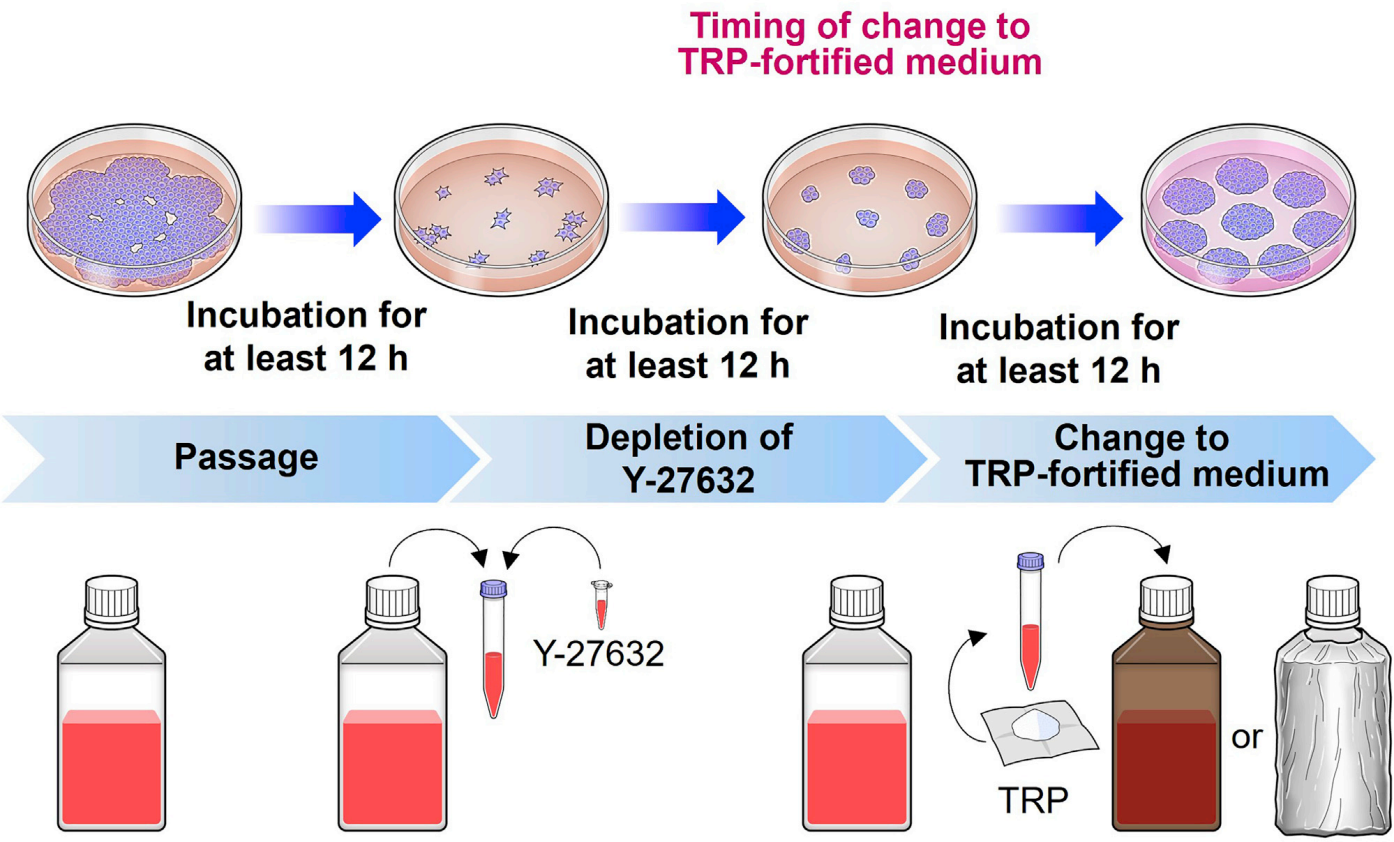
Flowchart guiding adjustment of hPSCs to TRP-fortified medium The switch to TRP-fortified medium from conventional TRP-concentration medium was performed after hPSCs were cultured in conventional TRP-concentration medium for at least 12 h.

***Alternatives:*** If cells were already cultured on a dish or plate containing mTeSR1, change the medium to TRP-fortified mTeSR1 on the next medium change day. If cells were grown in different media, they should be initially switched to mTeSR1 medium on the day after medium change, followed by TRP-fortified mTeSR1. If the cells cultured in non-fortified mTeSR1 are already confluent and ready for passage, they should not be passaged TRP-fortified mTeSR1 with Y-27632, as TRP fortification should commence at the time of medium change. If the cells were initially passaged using conventional mTeSR1 with Y-27632, and then the medium should be then changed to TRP-fortified mTeSR1 (Figure 2).

### Medium change of hPSCs

**Timing: 45 min**

In this step, hPSC culture medium is changed to Y-27632 depleted TRP-fortified medium.

***Note:*** As with the initial culture, the following steps should be undertaken under sterile conditions, except for assessments of the cells under the microscope.

12. After 1–2 days of incubation, place the required amount of TRP-fortified mTeSR1 solution in a conical tube covered by a shading sheet at room temperature (approximately 21°C) for approximately 30–60 min.
13. Observe cells in the dish or plate microscopically.

***Note:*** Observation of cells should be performed under an inverted microscope regularly prior to the timing of medium change, passage, or cryopreservation, particularly to assess the morphology indicative of differentiation and the presence or absence of contamination. The microscope should be thoroughly cleaned using 70% ethanol prior to and after use.

**CRITICAL:** Any signs suggestive of contamination, discontinue culture, and discard all concurrently cultured dishes and plates to prevent the dissemination of contamination in the laboratory.

14. Tilt the dish or plate, aspirate the culture medium, and change medium to TRP-fortified mTeSR1 solution (without Y-27632). Troubleshooting 3.

***Note:*** During medium change or passage, if possible, minimize the time of direct light exposure, which may be detrimental to cells, and reduce the light level in a sterile hood.

**Pause point:** Change the medium every other day with TRP-fortified mTeSR1 solution. Once cells are 60%–80% confluent, they should be passaged to facilitate maintenance of pluripotency and spontaneous differentiation. The cells were passaged on day 7.

### Passage of hPSCs

**Timing: 45–75 min**

In this step, hPSCs in TRP-fortified medium are passaged and is seeded with planned densities.

15. Prepare resources for passaging when cells are ready.
  a. Transfer the required volume of TRP-fortified mTeSR1 to a conical tube with 10 μM Y-27632 and keep it in a dark place at room temperature (approximately 21°C) for warming.
  b. Prepare Matrigel-coated dishes or plates as described previously, and replace the coating with TRP-fortified mTeSR1 with 10 μM Y-27632.
16. Observe cells in the dish or plate microscopically.
17. Tilt the dish or plate and aspirate medium.
18. Wash the dish or plate gently with PBS and aspirate the supernatant.
19. Add TrypLE Select for dissociation and the dish or plate was placed in an incubator at 37°C for approximately 3 min.

***Note:*** The amount of TrypLE Select depends on the surface area of the dish or plate and may need to be adjusted based on confluence. We typically added 2 mL to a 10-cm dish and 500 μL per well in a 6-well plate.

***Alternatives:*** Accutase can be used as an alternative to TrypLE Select. We typically added 2 mL to a 10-cm dish and 500 μL per well in a 6-well plate.

***Note:*** Observe the dish or plate to ensure that the cells are well-dissociated prior to the next step. Incubate for longer periods if the cells do not dissociate.

20. Add 10 mL or 2 mL per well of TRP-fortified mTeSR1 with Y-27632 to a 10-cm dish or 6-well plate, respectively. Then, gently detach the cells, and transfer all the cell suspensions to a conical tube using an electronic pipette controller.

***Alternatives:*** Cells can be collected directly using a 1,000 μL pipette tip after detachment by gentle pipetting. The collected cells were promptly transferred to a tube containing 10 mL TRP-fortified StemFit AK02N with Y-27632.

21. Centrifuge the tube for 3 min at 300 × *g.*
22. Aspirate the supernatant carefully to prevent suction of the cell pellet; mix 10 mL of normal mTeSR1 with 10 μM Y-27632 and gently stir cells, preferably using multiple bouts of suction; discharge contents using an electronic pipette controller with a sterile serological pipette.
23. Perform a cell count using Vi-CELL XR or equivalent, and assess cell density within the sampled solution and viability.
24. Seed cells onto prepared dishes or plates based on the planned cell density or split-cell ratio.

***Note:*** The preferred cell density depends on multiple variables, including the cell line, passage number, and adaptation to TRP-fortified medium. Several attempts are required to determine the optimal cell density or split ratio. We suggest initial seeding and culture with different cell densities using multiple dishes or plates and choose one of them that is approximately 60%–80% confluent at the time of passaging. In this experiment, 1.7 × 10^3^ cells per cm^2^ of cells were seeded.

25. Place the dishes or plates in an incubator at 37°C and gently move them side-to-side to evenly spread the cells without contamination.

**Pause point:** Continue culture using TRP-fortified mTeSR1 for at least two weeks to allow hPSCs to adapt to their growth environment. Troubleshooting 4.

### Cryopreservation of hPSCs

**Timing: 45–60 min**

In this step, hPSCs in TRP-fortified medium are cryopreserved.

Cryopreservation of hPSCs is recommended when the cells are adapted to TRP-fortified medium after a few passages, allowing easier access to cells that have an increased capacity to proliferate and to efficiently commence cell culture when another culture encounters problems, such as contamination or undesirable differentiation.

26. Prepare 10 mL of TRP-fortified mTeSR1 with 10 μM Y-27632 in a conical tube and keep it in the dark (the medium does not need to be warmed).
27. Prepare cryotubes and record data, such as date, cell line, passage number, TRP-fortification status, cell count, and medium used before freezing.
28. Repeat steps 16–21.
29. Centrifuge the tube for 3 min at 300 × *g.*
30. Aspirate the supernatant.
31. Add 500 μL of STEM-CELLBANKER per 1–5 × 10^6^ cells.

***Note:*** Cell density that is either too high or too low may cause decreased survival after thawing.

32. Gently disperse the cells by pipetting with a sterile 1,000 μL tip attached to a P1000 pipette.

***Note:*** The cells are very fragile at this point. Only three pipetting strokes are required to disperse the pellets. More than five strokes can decrease the viability.

33. Immediately after pipetting, 500 μL of cryopreservation solution should be transferred to each of the prepared cryotubes.
34. Place the cryotubes in BICELL freezing vessels.
35. Store the vessels in a −80°C freezer.

**Pause point:** Cryotubes should be removed from the vessels overnight (8–24 h) and stored in a freezer at −150°C for long-term preservation.

***Note:*** Frozen hPSCs can be recovered in steps 1–11, except using TRP-fortified mTeSR1 with Y-27632 as the culture medium from the start.

### Evaluation of hPSCs proliferation: Live-cell imaging

**Timing: 30–60 min**

In this step, hPSCs are evaluated in terms of proliferation.

IncuCyte ZOOM is used to determine proliferation rates by serially measuring the confluence for specified periods. This system enables maintenance of the ongoing culture of cells while comparing proliferation rates between TRP-fortified medium and conventional medium in an installed humidified 5% CO_2_ incubator at 37°C.

36. Turn on IncuCyte and establish a protocol for imaging, that includes setting up the type of plates and cells, scan mode, imaging intervals, and parameters for image processing.
37. Repeat steps 15–24, with the exception that plates that are compatible with the imaging system must be used instead of passaging onto 10-cm dishes.
38. After seeding, the plates should be gently moved side-to-side to evenly spread the cells without contamination, and gently but firmly placed in the instrument tray specific to the culture vessel.
39. Start imaging and change medium as required (Methods Video S1).

***Note:*** Avoid opening the drawer of IncuCyte during the first 24 h of culture, as cells are more susceptible to mechanical force shortly after passage.

40. When the acquisition protocol established during step 36 finishes, the data should be analyzed using MS-Excel. Troubleshooting 5.

Methods Video S1. Proliferation of hPSCs in TRP-fortified medium, related to step 39 The orange area indicates the area annotated by IncuCyte ZOOM.

### Evaluation of hPSCs pluripotency: Flow cytometry

**Timing: 60–90 min**

In this step, hPSCs are evaluated in terms of pluripotency using cytometry.

Flow cytometry of hPSCs cultured in TRP-fortified medium should be performed after multiple passages to assess their pluripotency using hPSCs grown in conventional medium as a control. Three antibodies (anti-REA, anti-SSEA4, and anti-TRA-1-60) were used for this assay. The Gallios flow cytometer and its software, Kaluza and FlowJo, were used for measurement and analysis.

41. Prepare hPSCs that are approximately 40%–60% confluent grown on 10-cm dishes or 6-well plates.
42. Repeat steps 16–21 to perform cell counts.
43. Transfer the medium containing 3 × 10^6^–6 × 10^6^ cells per cell line to another conical tube.

***Note:*** When performing flow cytometry to compare pluripotency between hPSCs in TRP-fortified medium and hPSCs in conventional medium, the same number of cells should be sampled from each condition.

44. Centrifuge the tube for 3 min at 300 × *g.*
45. Aspirate The supernatant and add 300 μL of a solution containing 2% FBS in PBS.
46. Divide the solution into three aliquots of 100 μL each in a 1.5 mL microtube.
47. Add 10 μL of each antibody to a separate microtube.

**Pause point:** Place the microtubes in the dark on ice for 30 min.

48. Add 1 mL of 2% FBS to each microtube and centrifuge for 3 min at 1,250 × *g* (or 5 min at 300 × *g*).
49. Using a pipette tip, aspirate supernatant.
50. Add 500 μL of 2% FBS in PBS to each of the microtubes.
51. Dispense the solutions to a separate test tube by filtering them through a cell strainer cap.
52. Run the flow cytometer.

### Evaluation of hPSCs pluripotency: Immunocytochemistry

**Timing: 2–3 h**

hPSCs are evaluated in terms of pluripotency using immunocytochemistry.

Immunocytochemistry of hPSCs cultured in TRP-fortified medium is recommended to assess their pluripotency, and should be performed together with hPSCs grown in conventional medium as a control. Three pluripotency markers were used in this assay (OCT-4, NANOG, and SSEA4). Consult the manufacturer’s instructions for details regarding the appropriate dilution ratio for each antibody, and ensure that the secondary antibodies correspond to the primary antibodies used. The fluorescence microscope BZ-X710 and its accompanying software, BZ-X Viewer and BZ-X Analyzer, were used for analysis.

53. Prepare hPSCs at 40%–60% confluency on 6-well plates.

***Note:*** If three pluripotency markers are tested, a minimum of three wells are required for immunocytochemistry per cell line.

54. Aspirate medium.
55. Wash with 2 mL of PBS per well and aspirate.
56. Fix Cells by adding 1–1.5 mL of 4% paraformaldehyde per well and refrigerated at 4°C.

**Pause point:** Place the plates in a refrigerator at 4°C for 30–60 min.

57. Aspirate 4% paraformaldehyde.
58. Wash with 2 mL of PBS per well and aspirate (twice).
59. Add 1 mL of 0.1% Triton X-100 per well to permeabilize cells and leave at room temperature (approximately 21°C) for 1–15 min.
60. Aspirate 0.1% Triton X-100.
61. Wash with 2 mL of PBS per well, and aspirate.
62. Add approximately 1.5–2 mL of ImmunoBlock per well.

**Pause point:** Blocking can be performed at room temperature (approximately 21°C) for 30 min to 1 h or at 4°C overnight (8–24 h).

63. Dilute the primary antibodies with ImmunoBlock in separate conical tubes (1 mL per well).
64. Aspirate ImmunoBlock from the plates and add diluted primary antibodies.

**Pause point:** Place the plates in a refrigerator at 4°C overnight (8–24 h).

65. Prepare the corresponding secondary antibodies diluted with ImmunoBlock in separate conical tubes (1 mL per well is required).
66. Wash with 5 mL of PBS per well and aspirate (twice).
67. Add diluted secondary antibodies to the plates.

**Pause point:** Place the plates in the dark at room temperature (approximately 21°C) for 2 h.

68. Wash with 5 mL of PBS per well and aspirate (twice).
69. Prepare 2 mL of 0.02 mg/mL Hoechst 33342 by mixing 1.998 mL of PBS and 0.001 mL of 10 mg/mL Hoechst 33342.
70. Add 1 mL of 0.02 mg/mL Hoechst 33342 per well and the plates were left in the dark at 20°C for 30 min.
71. Wash with 5 mL of PBS per well and aspirate (twice).
72. Add 1.5–2 mL of ImmunoBlock per well.
73. Image cells using a fluorescence inverted microscope suitable for immunohistochemistry.

***Note:*** If imaging is to be performed later, the plates can be stored in the dark place at 4°C for up to one week. Wrapping with a shade sheet to minimize light exposure is recommended.

## Expected outcomes

By applying this method, hPSCs were able to grow efficiently through multiple passages, with an increase in the 10-week cumulative cell counts ranging from 5- to 17.5-fold after the cells had adapted to the new medium. hPSCs proliferate for a long period without karyotypic changes (Someya et al., 2021).

**Figure 3:**
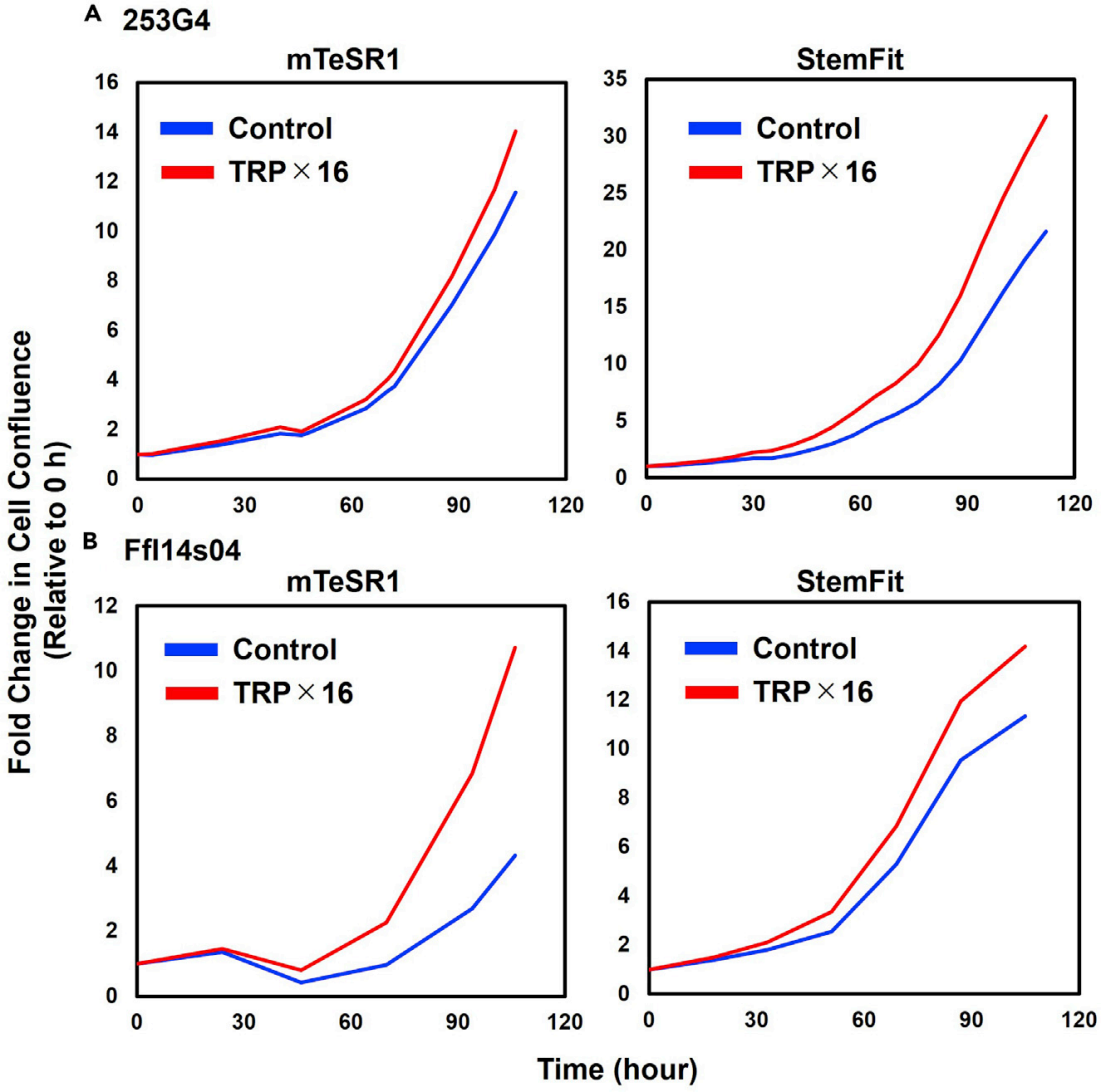
Proliferation of hPSCs maintained in TRP-fortified medium Cell confluence after 110 h of hPSC culture, with TRP-fortification occurring 24 h after seeding. The measurements of the cell area started at t = 0, which was defined as the time of the initial medium exchange to the TRP-fortified (16-fold TRP concentration) medium.

TRP fortification showed a consistent improvement in proliferation across multiple cell lines, including 253G4 and FfI14s04 for hiPSCs, suggesting that this phenomenon is not cell-specific (Figure 3, Methods video S1). For basement membrane matrices other than Matrigel, we tested iMatrix-511, a chemically defined, xeno-free recombinant human laminin substrate, which also showed enhanced proliferation after TRP fortification, implying that the increased proliferation was not dependent on the components of the basement membrane matrix.

**Figure 4:**
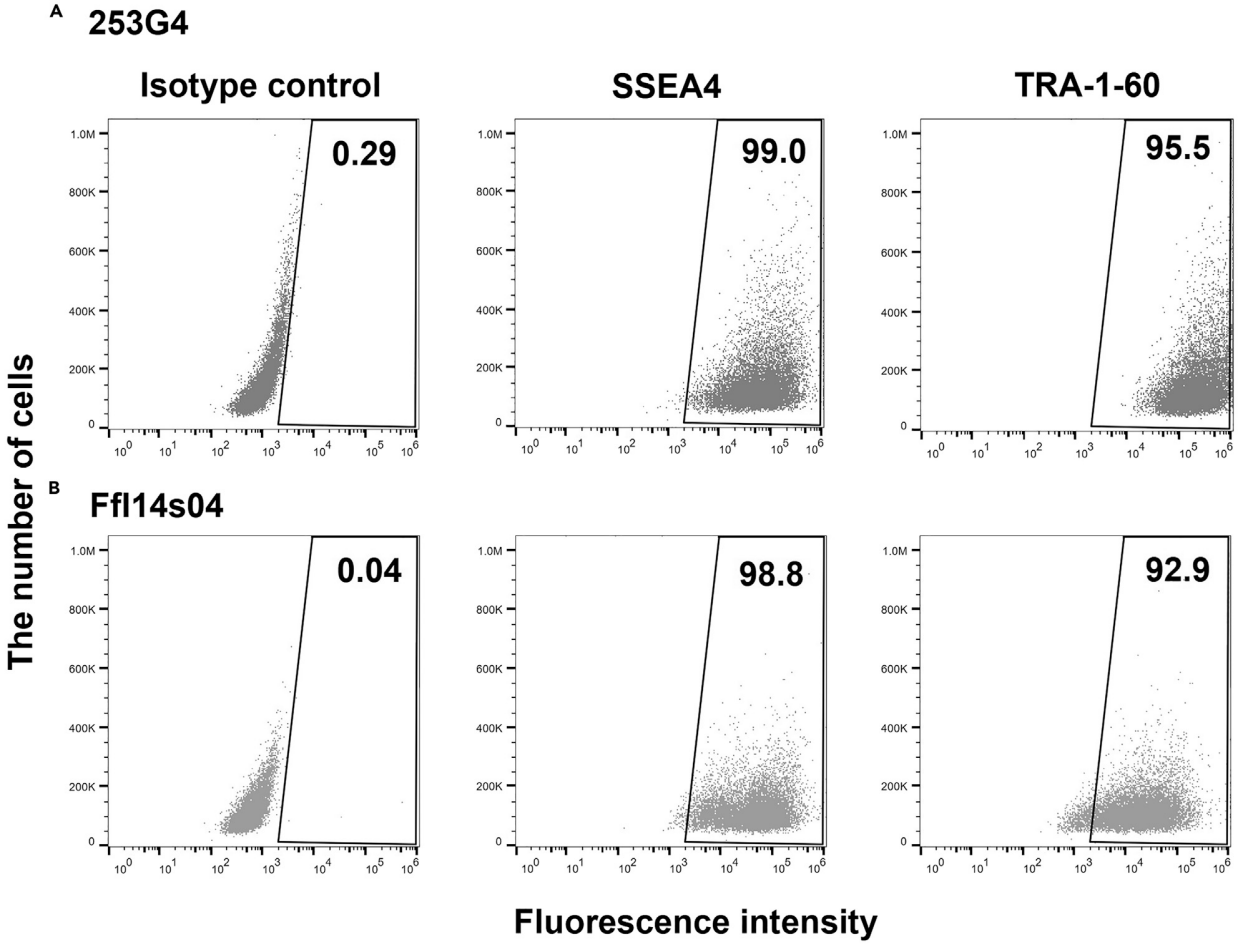
Population of hPSCs expressing pluripotency markers (A and B) Population of hPSCs expressing the pluripotency markers SSEA4 and TRA-1-60 in (A) 253G4 and (B) FfI14s04 lines.

**Figure 5:**
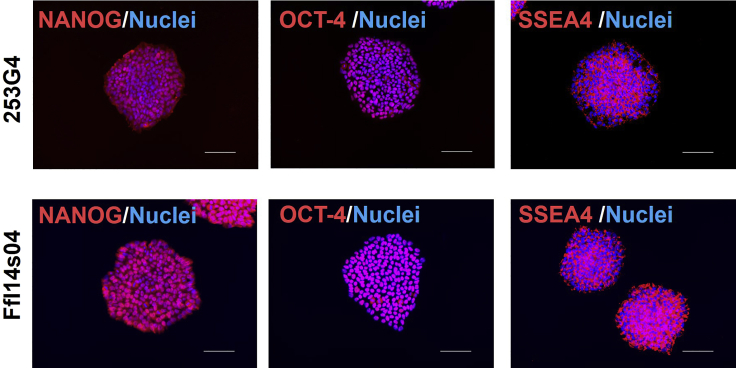
Representative images of hPSCs maintained in TRP-fortified medium stained for pluripotency markers HiPSCs (253G4 and FfI14s04) expanded in TRP-fortified medium were fixed and evaluated by immunocytochemistry. Red indicates of anti-OCT4 antibody, anti-NANOG antibody, or anti-SSEA4 staining. Blue indicates Hoechst Staining. Scale bar, 100 μm.

Pluripotency was assessed by multimodal analysis involving flow cytometry detection of SSEA4 and TRA-1-60 (Figure 4), as well as immunocytochemistry screening for OCT-4, NANOG, and SSEA4 (Figure 5), all of which showed a high level of pluripotency, demonstrating that hPSCs grown in TRP-fortified medium retained pluripotency after a long period.

## Limitations

Although TRP-fortified mTeSR1 medium was effective in multiple hPSC lines, including 253G4 and FfI14s04, whether enhanced proliferation is universal for all hPSC lines remains unclear. We validated that increased cell growth is observed in mTeSR1 and StemFit AK02N when supplemented with TRP; however, whether this applies to all available hPSC growth media remains unclear. The capacity of cells to adapt to TRP-fortified media and the number of passages required for cell adaptation may vary among different cell lines. Owing to the photodegradation of TRP, the degree of proliferation may be influenced by environmental factors, such as lighting in the laboratory and sterile hood, and time of exposure, which in turn may depend on how efficiently each step is performed.

## Troubleshooting

### Problem 1

TRP does not dissolve in mTeSR1.

### Potential solution

Ensure that the TRP used is of cell culture grade, handled with caution, and not contaminated with other substances. If a large mass of TRP remained at the bottom of the container after the addition of mTeSR1 basal medium, gently shake the container multiple times to help dissipate the mass (firmly close the lid beforehand to prevent spillage). If the dissolution process is slow, the amount of basal medium increases. We do not recommend leaving the solution at room temperature (approximately 21°C) for long periods or warming the solution to a 37°C water bath, as this may lead to TRP and medium deterioration. We also suggest not using solvents other than the basal medium (such as Milli-Q water or DMSO) to dissolve TRP, as they cause overall dilution of the culture medium and may have potentially deleterious effects in long-term culture (step 4).

### Problem 2

Detachment of colonies from the dish is observed.

### Potential solution

Colony detachment is often observed when the basal medium is changed. Re-validation of the Matrigel dilution ratio is required. If detachment is still observed, testing other extracellular matrices is required. In this case, validation of the use of iMatrix-511 is our first recommendation (step 8).

### Problem 3

Significant cell death is seen, or cells do not survive after initial culture.

### Potential solution

If frozen cells had been previously cultured in a different medium, thaw the cells initially in the same medium supplemented with Y-27632. After 1–2 days, change the medium to mTeSR1 without TRP-fortification until the cells proliferated and reached 60%–80% confluence. Cells were passaged as previously described for mTeSR1 (without TRP-fortification) with Y-27632, and after 1–2 days, the medium was changed to TRP-fortified mTeSR1. A few passages without TRP fortification may be required until the cells are fully adapted to mTeSR1, as the cells likely do not survive well with TRP fortification unless they are adapted to the medium. If cells still do not survive, check for signs of contamination, differentiation, or karyotype abnormality; alternatively, choose cells with fewer passages if available (step 14).

### Problem 4

Cell growth is slow after TRP-fortification.

### Potential solution

Slower growth is commonly observed in the first few passages, especially when passaging with TRP-fortified medium for the first time. Adaptation to TRP-fortified medium may vary from cell line to cell line, and some cell lines may require longer time for cell adaptation. Adjust The split ratio so that the confluence was 60%–80% at the time of passage, as a too small or too large cell density is detrimental to cell growth. Overconfluent cells may lose pluripotency. When compared to the control, the same cell line and passage number were used. In addition, we ensured that the TRP-fortified basal medium was the same as that used before using the TRP-fortified basal medium. Pluripotent stem cells must be acclimatized to any new basal medium for at least two passages (step 25).

### Problem 5

Results obtained using IncuCyte are inaccurate.

### Potential solution

The IncuCyte system was calibrated. Review the configurations of the system and software, including the setup for scan mode and job analysis. Consider a whole-well scan mode instead of a standard scan mode and refine the job analysis to allow better recognition of phase-contrast images. Multiple attempts may be required to obtain an optimal configuration before a formal experiment can be conducted. For accurate results, avoiding the use of high-number plates (for example, 24-well or more plates) decreases the accuracy. The cells were evenly distributed during seeding. For a 6-well plate, we suggest replacing Matrigel with a small volume of growth medium with Y-27632, initially (such as 500 μL per well), and then dispensing the diluted solution with a larger volume (such as 1.5 mL per well) containing a designated number of cells to avoid seeding directly from a high-density suspension, which may cause more variation in the initial distribution (step 40).

## Resource availability

### Lead contact

Further information and requests for resources and reagents should be directed to and will be fulfilled by the lead contact Shugo Tohyama (shugotohyama@keio.ac.jp).

### Materials availability

This study did not generate new unique reagents.

## Data Availability

There is no dataset and/or code associated with the article.
